# An Induced Pluripotent Stem Cell-Derived Human Blood–Brain Barrier (BBB) Model to Test the Crossing by Adeno-Associated Virus (AAV) Vectors and Antisense Oligonucleotides

**DOI:** 10.3390/biomedicines11102700

**Published:** 2023-10-04

**Authors:** Jamuna Selvakumaran, Simona Ursu, Melissa Bowerman, Ngoc Lu-Nguyen, Matthew J. Wood, Alberto Malerba, Rafael J. Yáñez-Muñoz

**Affiliations:** 1 AGCTlab, Centre of Gene and Cell Therapy, Department of Biological Sciences, School of Life Sciences and the Environment, Royal Holloway University of London, Egham TW20 0EX, UK; jamuna.selvakumaran@rhul.ac.uk (J.S.); simona.ursu@uni-ulm.de (S.U.); 2 School of Medicine, Keele University, Staffordshire ST4 7QB, UK; m.bowerman@keele.ac.uk; 3 Wolfson Centre for Inherited Neuromuscular Disease, RJAH Orthopaedic Hospital, Oswestry SY10 7AG, UK; 4 Gene Medicine Laboratory for Rare Diseases, Centre of Gene and Cell Therapy, Department of Biological Sciences, School of Life Sciences and the Environment, Royal Holloway University of London, Egham TW20 0EX, UK; ngoc.lu-nguyen@rhul.ac.uk (N.L.-N.); alberto.malerba@rhul.ac.uk (A.M.); 5 Department of Paediatrics, Institute of Developmental and Regenerative Medicine (IDRM), University of Oxford, Oxford OX3 7TY, UK; matthew.wood@idrm.ox.ac.uk; 6 MDUK Oxford Neuromuscular Centre, University of Oxford, Oxford OX3 9DU, UK

**Keywords:** human blood–brain barrier, brain microvascular endothelial cells, induced pluripotent stem cells, trans-endothelial electrical resistance, gene therapy, spinal muscular atrophy, antisense oligonucleotides, phosphorodiamidate morpholino oligomers, adeno-associated virus vectors

## Abstract

The blood–brain barrier (BBB) is the specialised microvasculature system that shields the central nervous system (CNS) from potentially toxic agents. Attempts to develop therapeutic agents targeting the CNS have been hindered by the lack of predictive models of BBB crossing. In vitro models mimicking the human BBB are of great interest, and advances in induced pluripotent stem cell (iPSC) technologies and the availability of reproducible differentiation protocols have facilitated progress. In this study, we present the efficient differentiation of three different wild-type iPSC lines into brain microvascular endothelial cells (BMECs). Once differentiated, cells displayed several features of BMECs and exhibited significant barrier tightness as measured by trans-endothelial electrical resistance (TEER), ranging from 1500 to >6000 Ωcm^2^. To assess the functionality of our BBB models, we analysed the crossing efficiency of adeno-associated virus (AAV) vectors and peptide-conjugated antisense oligonucleotides, both currently used in genetic approaches for the treatment of rare diseases. We demonstrated superior barrier crossing by AAV serotype 9 compared to serotype 8, and no crossing by a cell-penetrating peptide-conjugated antisense oligonucleotide. In conclusion, our study shows that iPSC-based models of the human BBB display robust phenotypes and could be used to screen drugs for CNS penetration in culture.

## 1. Introduction

The blood–brain barrier (BBB) is primarily composed of highly specialised brain microvascular endothelial cells (BMECs) sharing the basal lamina with pericytes and end-processes of astrocytes [1]. Although the BBB successfully controls the exchange of molecules and cells between the brain and the blood, it also prevents the crossing of therapeutics that would be beneficial for the treatment of diseases affecting the central nervous system (CNS). Indeed, drug development for the majority of CNS disorders has failed due to lack of penetration of therapeutic candidates across the BBB [2]. Efforts to develop in vitro BBB models that mimic the complex in vivo properties have been ongoing for many decades. Early in vitro models were mainly produced using primary BMECs from animal brain tissue of different origins [3]. The next generation of BBB models included co-cultures of primary BMECs from animal brain tissue with different combinations of other BBB cells, such as pericytes and astrocytes [4]. BBB models derived from animal tissues have been very useful to characterise generic BBB properties, but high interspecies variability and ethical issues are promoting the research into alternative and sustainable models. Human models of the BBB have been prepared using brain tissue biopsies [5] or immortalised endothelial cell lines [5,6]. However, the most commonly used model, based on the immortalised human cerebral microvascular endothelial cell line hCMEC/D3, has several limitations, including low trans-endothelial electrical resistance (TEER), reflecting low barrier tightness [7]. Transcriptional profiling has shown reduced expression of tight junction proteins and glucose transporter 1 (GLUT–1) in hCMEC/D3 cells, in contrast to human primary BMECs [8].

Recent advances in iPSC technologies have made it possible to reliably produce cell lineages of interest in culture. Development of a reproducible protocol for differentiation of iPSCs into BMECs by Shusta’s group [9] has enabled the successful production of human models of the BBB [10]. The tight junction between BMECs is the most recognised phenotype of the BBB and is defined by high trans-endothelial electrical resistance (TEER) and low permeability to para-cellular markers [11]. Water soluble molecules can only cross the BBB by the para-cellular route and tight junctions play an important function in restricting para-cellular permeability to even small ions such as Na^+^ and Cl^−^ [1]. Permeability to para-cellular markers is widely used to characterise iPSC-derived models of the BBB [12,13]. The most commonly used para-cellular fluorescent tracers are lucifer yellow, LY, and sodium fluorescein, Na-F [14].

Gene therapy approaches for treating human disease have recently accomplished significant successes. In particular, the development and marketing approval of antisense therapeutics (nusinersen, brand name Spinraza) and adeno-associated virus (AAV) gene therapy (onasemnogene abeparvovec, Zolgensma) for the treatment of spinal muscular atrophy (SMA) have represented an important milestone [15]. Nusinersen exemplifies the difficulty of delivering drugs to the CNS as it is an antisense oligonucleotide based on the 2′-MOE chemistry that does not cross the BBB and requires repeated administration by intrathecal injection [16]. Antisense oligonucleotides made with other chemistries have similar issues, and modifications to improve CNS delivery, for instance, peptide conjugation, have been designed for several diseases, including SMA [17,18]. Phosphorodiamidate morpholino oligomers (PMOs) are a type of antisense oligonucleotide which can be suitably modified by peptide conjugation. Systemically administered PMO internalising peptide 6a (Pip6a)-conjugated PMOs have been shown to have activity in the CNS of a mouse model of SMA [19]. Similarly, viral vectors able to cross the BBB are of significant interest. Attention has recently focused on AAV vectors, with many serotypes that have been tested for suitability to cross the BBB in animal models in vivo. AAV serotype 9 (AAV9) is particularly proficient in this respect [20,21] and has been used for transgene delivery in the context of SMA [22] (now on the market as Zolgensma) and is in clinical trials for other diseases [23].

In vitro models to screen and investigate BBB crossing by such potential therapeutics would significantly aid development and contribute to reducing reliance on animal experiments in the pre-clinical settings. Patient-specific iPSC-derived BBB models have been very useful to understand mechanisms of disease and screening therapeutics [24]. Although co-culture and microfluidic systems can increase barrier tightness, they are complex, not easy to reproduce, labour intensive and time-consuming. Here, we have investigated monoculture models of human BBB and explored the reproducibility of BBB models produced from three different fibroblast-derived iPSC clones, using hCMEC/D3 cells as a benchmark for comparison. We validated the BBB models using immunocytochemistry, flow cytometry, TEER and permeability to fluorescent tracers, and then used them to test the crossing of AAV8 and AAV9 as well as peptide-conjugated oligonucleotides. We show that the tightness of the iPSC-derived BMEC models depends on the iPSC clone and that a tight BBB can be achieved with BMECs alone. We furthermore show cell line- and time-dependent BBB penetration by AAV9. Our results overall present a simple, scalable, robust, and reproducible iPSC-derived BBB model that has features superior to the commonly used human hCMEC/D3 system to test therapeutics for CNS penetration.

## 2. Materials and Methods

### 2.1. Cell Culture and iPSC Differentiation to BMECs

Unless otherwise stated, all chemicals were purchased from Merck (Dorset, UK). hCMEC/D3 cells were maintained on 0.1 mg/mL rat tail collagen I (Thermo Fisher Scientific, Cheshire, UK)-coated tissue culture flasks in endothelial cell growth medium (EGM-2 MV Bullet kit, Lonza, Slough, UK). Coverslips, culture plates and Transwell^®^ permeable polyester inserts (Corning, Flintshire, UK) were coated with collagen I before seeding hCMEC/D3 cells. Human fibroblast-derived iPSC clone 4603 (reprogrammed in house using retroviral vectors) and clones 19-9-7T and AD3-CL1 (reprogrammed using episomal vectors) were maintained on Matrigel (Corning, Flintshire, UK) in mTeSR1 medium (STEMCELL Technologies, Cambridge, UK). iPSCs were differentiated to brain BMECs using the protocol by Lippmann et al. [25]. In order to differentiate the iPSCs, cells were seeded on Matrigel in mTeSR1 medium and expanded for 3 days before switching to unconditioned medium (UM) without basic fibroblast growth factor (bFGF) for 5 days. Cells were cultured in human endothelial serum-free medium (hESFM; Thermo Fisher Scientific, Cheshire, UK) supplemented with 20 ng/mL bFGF (R&D Systems, Abingdon, UK), 1% platelet-poor plasma derived bovine serum (PDS, Alfa Aesar, Haverhill, MA, USA) and 10 μM all-trans retinoic acid for an additional 2 days. Culture plates, inserts and coverslips were incubated with a mixture of 1 mg/mL collagen IV, 0.5 mg/mL fibronectin and water in a ratio of 4:1:5 for a minimum of 4 h at 37 °C. Cells were then dissociated with accutase (Thermo Fisher Scientific, Cheshire, UK) and seeded onto coverslips and inserts coated with collagen IV and fibronectin. Cells were grown on the plates, coverslips or inserts in the same medium for 24 h before changing it with 1% PDS for a further 24 h.

### 2.2. Immunocytochemical Analysis of Tight-Junction Protein

Cells were fixed with 4% paraformaldehyde for 15 min at room temperature and washed twice with ice-cold phosphate buffered saline (PBS, Thermo Fisher Scientific, Cheshire, UK), then permeabilised with 0.25% Triton X-100 for 10 min at room temperature and washed with PBS three times for 5 min. This was followed by blocking in 1% bovine serum albumin (BSA) in PBS-Tween for 30 min at room temperature, and incubation with Alexa Fluor 488 conjugated occludin or claudin–5 monoclonal antibody (Thermo Fisher Scientific, Cheshire, UK, catalogue number: 331588 and 352588, respectively) diluted 1:100 with 1% BSA in PBS-Tween for 1 h at room temperature in the dark. Cells were washed three times with PBS for 5 min and nuclei were stained with 1 μg/mL DAPI for 1 min at room temperature. Finally, cells were washed with PBS before mounting the slides. Images were captured with a Zeiss Axio Observer D1 fluorescence microscope using Zen software (Zeiss, Cambridge, UK).

### 2.3. Flow Cytometry

Cells were dissociated with accutase, spun down and washed with PBS. FACS buffer (PBS containing 1% BSA and 0.1% sodium azide) was used to resuspend the cell pellet. Around 500,000 cells were incubated with 1:10 diluted GLUT–1 antibody conjugated to fluorescein (R&D Systems, Abingdon, UK, catalogue number: FAB1418F) and PECAM–1 antibody conjugated to PE (BD Biosciences, Berkshire, UK, catalogue number: 340297) for 30 min at room temperature. Cells were washed twice with FACS buffer, resuspended in 200 μL FACS buffer and analysed on a BD FACS Canto II flow cytometer (BD, Berkshire, UK). GLUT–1^+^ and PECAM–1^+^ cells were analysed with reference to unstained cells.

### 2.4. TEER Measurements

Transwell^®^ permeable polyester inserts (Corning, Flintshire, UK) with 1.12 cm^2^ area and 0.4 μm pore size were coated with either collagen I (hCMEC/D3) or collagen IV and fibronectin (iPSC-derived BMECs). Cells were seeded at a density of 900,000 cells/cm^2^ for 48 h. The TEER of the cell monolayer and the inserts coated with collagen IV and fibronectin or collagen I was measured using an EVOM voltohmmeter with STX2 electrodes (World Precision Instruments, Hertfordshire, UK). The resistance of the coated inserts was subtracted from the resistance of the cell monolayer and then multiplied by the surface area of the insert (1.12 cm^2^) to calculate the TEER in Ωcm^2^. Cells were equilibrated to room temperature for 20 min before the TEER measurements and allowed to recover for at least 30 min before further experimentation.

### 2.5. Permeability of Fluorescent Tracers

Permeability assays were performed using cells in Transwell^®^ inserts 30 min after TEER measurements. Stocks were prepared by dissolving LY and Na-F to a concentration of 0.1 mg/mL in Ringer HEPES buffer (150 mM sodium chloride, 2.2 mM calcium chloride, 0.2 mM magnesium chloride, 5.2 mM potassium chloride, 2.8 mM glucose, 6 mM sodium bicarbonate and 5 mM 4-(2hydroxyethyl)-1-piperazineethanesulfonic acid, pH 7.4). Medium was aspirated from both apical and basal chambers. Ringer HEPES buffer was pre-warmed to 37 °C, 1 mL of Ringer HEPES buffer was added to the basal chamber and 0.5 mL of stock (0.1 mg/mL) of LY or Na-F in Ringer HEPES buffer was added to the apical chamber. Cells were incubated at 37 °C for 60 min. A total of 150 μL from each basal chamber was transferred to a 96 well plate, along with 0.1 mg/mL stock solutions of LY and Na-F and a blank solution of Ringer HEPES buffer. Fluorescence intensity was measured immediately using a SpectraMax Gemini XS microplate reader (Molecular Devices, Berkshire, UK). The percentage of permeability was calculated from the relative fluorescence units using the following formula:

$$\%\;Permeability=\frac{Sample-Blank}{Stock-Blank}\times 100$$

The values obtained were doubled to compensate for the 2-fold increase in volume in the basal chamber compared to the apical chamber.

### 2.6. AAV8 and AAV9 Preparation and Crossing of the BBB

ssAAV9-CBA-eGFP was custom-produced by Atlantic Gene Therapies. ssAAV8-Spc512-eGFP and scAAV9-CMV-eGFP vectors were produced in house by transfection of adherent HEK293T/c17 cells in Corning roller bottles (Corning, Flintshire, UK) with a two-plasmid system using polyethylenimine (PEI). Cells and supernatant were harvested 3 days post-transfection and centrifuged at 3000× *g* for 10 min. The AAV vector in the supernatant was concentrated by overnight precipitation at 4 °C with a final concentration of 8% (*w/v*) Poly(ethylene glycol) 8000 (PEG 8000)/0.5 M NaCl solution. The precipitated vector was then centrifuged at 2500× *g*, at 4 °C for 30 min. The vector-containing cell pellet and the precipitated vector pellet were resuspended in lysis buffer (50 mM Tris-HCl pH8.5, 150 mM NaCl, and 2 mM MgCl_2_), combined and subjected to three freeze/thaw cycles to lyse the cells. The virus particles were purified on an iodixanol gradient of layered 60%, 40%, 25%, and 15% iodixanol (OptiPrep, Dorset, UK) and centrifuged at 350,000× *g*, at 18 °C for 2 h. The AAV vector-enriched, 40% iodixanol layer was desalted and concentrated using 1X PBS-MK (PBS, 1 mM MgCl2, 2.5 mM KCl and 0.001% Poloxamer 188 Non-ionic Surfactant (Thermo Fisher Scientific, Cheshire, UK, catalogue number: 24040032) and Amicon Ultra-15 (Merck, Dorset, UK, catalogue number: UFC910024). Several centrifugation steps were carried out at 4500× *g*, at 4 °C for 20 min per step until the volume was reduced to 250 µL. The AAV vector genome concentration, expressed in vector genomes (vg)/mL, was determined using real-time quantitative PCR (qPCR).

To assess the crossing of AAV vectors through the iPSC-derived BBB models, differentiated cells were seeded on Transwell^®^ inserts at a density of 900,000 cells/cm^2^ and cultured for 48 h, after which 7.7 × 10^10^ vector genome (vg) units of ssAAV9-CBA-eGFP were added to the apical chamber of the Transwell^®^. Next, 20 μL samples were taken from the basal chamber at 4, 24, 48 and 72 h post-vector treatment. To compare the crossing of AAV8 and AAV9 vectors, 7.7 × 10^10^ vg units of ssAAV8-Spc512-eGFP or scAAV9-CMV-eGFP were added to the apical chamber of the Transwell^®^ using only 4603-derived BMECs. At 72 h post-vector treatment, 20 μL samples were taken from the basal chamber. DNA was extracted using DNeasy kit (Qiagen, Manchester, UK) following DNase I (Qiagen, Manchester, UK) and proteinase K (Qiagen, Manchester, UK) treatment. qPCR was performed using 500 nM primers for CBA promoter in ssAAV9-CBA-eGFP (FWD CBA primer: AACGCCAATAGGGACTTTCC, REV CBA primer: GTCAATAGGGGGCGTACTTG) or primers for the inverted terminal repeat (ITR) in ssAAV8-Spc512-eGFP and scAAV9-CMV-eGFP (FWD ITR primer: GGAACCCCTAGTGATGGAGTT, REV ITR primer: CGGCCTCAGTGAGCGA) and SensiMix™ SYBR^®^ No-ROX Kit (Meridian Bioscience, London, UK). DNA from viral stock was serially diluted and 5 µL of each dilution was used in qPCR to prepare the standard curve.

### 2.7. Assay of BBB Permeability to PMOs

For the PMO permeability studies, 4603-derived BMECs were seeded on Transwell^®^ inserts at a density of 900,000 cells/cm^2^ and SMA type I fibroblasts were cultured in the basal chamber of the Transwell^®^ at a density of 26,000 cells/cm^2^ for 48 h. PMOs enhancing *SMN2* pre-mRNA exon 7 inclusion [19] were incubated for 30 min at 37 °C and diluted in hESFM + 1%PDS, and 10 μM PMO was added to the apical chamber of the Transwell^®^ insert with BMECs. As a control, 2.5 μM PMO was added directly to the SMA type I fibroblast cells. The SMA type I fibroblasts were harvested 24 h later and RNA was extracted using the RNeasy mini kit (Qiagen, Manchester, UK). cDNA was synthesised using High-Capacity cDNA Reverse Transcription Kit (Thermo Fisher Scientific, Cheshire, UK) with 1 μg of RNA. qPCR was performed using SensiMix™ SYBR^®^ No-ROX Kit (Meridian Bioscience, London, UK) with 20 ng of cDNA per reaction in a total volume of 20 μL. Primers for *GAPDH*, ∆7 *SMN2* and full-length *SMN2* were used at a concentration of 500 nM (*GAPDH* FWD: AAAGGGTCATCATCTCCGCC, *GAPDH* REV: ACTGTGGTCATGAGCCCTTC, ∆7 *SMN2* FWD: TGGACCACCAATAATTCCCC, ∆7 *SMN2* REV: ATGCCAGCATTTCCATATAATAGCC, FL *SMN2* FWD: GCTTTGGGAAGTATGTTAATTTCA, FL *SMN2* REV: CTATGCCAGCATTTCTCCTTAATT). Data were normalised to the housekeeping gene *GAPDH*, standardised to levels in mock fibroblasts and analysed using the 2^−∆∆CT^ method. Data were expressed as full-length to ∆7 *SMN2* mRNA ratio.

### 2.8. Statistics

Experiments were performed in triplicate as a minimum. Data are presented as mean ± standard error of the mean (SEM). Graphpad Prism Version 6.07 (Boston, MA, USA) was used to analyse data. Statistical significance was analysed by using one-way or two-way analysis of variance (ANOVA) followed by Tukey post-hoc analysis. *p* values of less than 0.05 were considered statistically significant.

## 3. Results

### 3.1. Characterisation of BMECs Using Immunocytochemistry and Flow Cytometry

**Figure 1:**
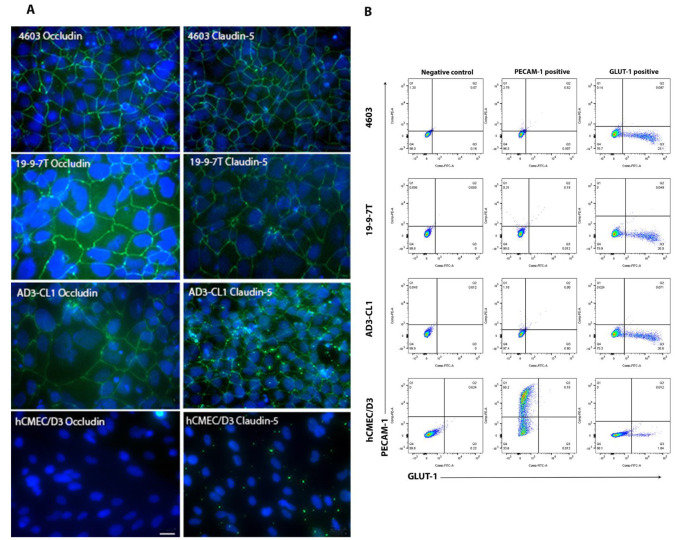
Immunocytochemical and flow cytometry detection of BBB proteins occludin, claudin–5, PECAM–1 and GLUT–1. (**A**) Presence of occludin (green, **left** column) and claudin–5 (green, **right** column) in iPSC-derived BMECs contrasts with lack of these proteins in hCMEC/D3. DAPI nuclear stain is overlaid in blue. Scale bar 20 µm. (**B**) Flow cytometry dot-plots of fluorescein-conjugated GLUT–1 and PE-conjugated PECAM–1 in iPSC-derived BMEC and hCMEC/D3. Cells were stained with anti-PECAM–1 or anti-GLUT–1 and analysed using flow cytometry. Unstained cells were used to gate the negative population (**left** column). GLUT–1 is present in a considerable fraction of iPSC BMECs, but only 0.3–3% of them display PECAM–1. In contrast, two thirds of hCMEC/D3 produce PECAM–1 but less than 2% are positive for GLUT–1.

The BBB phenotype is characterised by very tight junctions between BMECs. Several proteins are involved in forming the tight junctions and the presence of two of them, occludin and claudin–5, was assessed using immunocytochemistry (Figure 1A). Three different iPSC clones (4603, 19-9-7T and AD3-CL1) were differentiated into BMECs, seeded onto collagen IV and fibronectin-coated coverslips at an optimal density of 900,000 cells/cm^2^ and cultured for 48 h. hCMEC/D3 cells were similarly seeded on collagen I-coated coverslips and kept for 48 h. BMECs derived from iPSC clones 4603 and 19-9-7T exhibited uniform expression of both claudin–5 and occludin. Although BMECs derived from AD3-CL1 showed less widespread expression of both claudin–5 and occludin, this was still in contrast with hCMEC/D3, where these proteins were undetectable.

GLUT–1 is responsible for glucose transport to the brain and is considered a key marker of BMECs. Platelet Endothelial Cell Adhesion Molecule–1 (PECAM–1) is involved in angiogenesis, leukocyte migration and integrin activation. The analysis of both proteins can easily be performed using flow cytometry and we used this technique to further characterise the iPSC-derived BMECs (Figure 1B). Cells were dissociated with accutase and incubated with PECAM–1 or GLUT–1 primary antibodies. Unstained cells were used to gate the negative population. For all iPSC-derived BMECs, more than 20% of cells were positive for GLUT–1, whereas around 2% of hCMEC/D3 were positive for this protein. Furthermore, around 66% of hCMEC/D3 cells produce PECAM–1, while in iPSC-derived BMECs, production varies between 0.35% and 3% of cells depending on the clone. These data contrast with other pluripotent cell-based studies, where the presence of PECAM–1 and elevated levels of GLUT–1 were used as characteristic of BBB models.

### 3.2. TEER-Based Evaluation of the BBB Models

**Figure 2:**
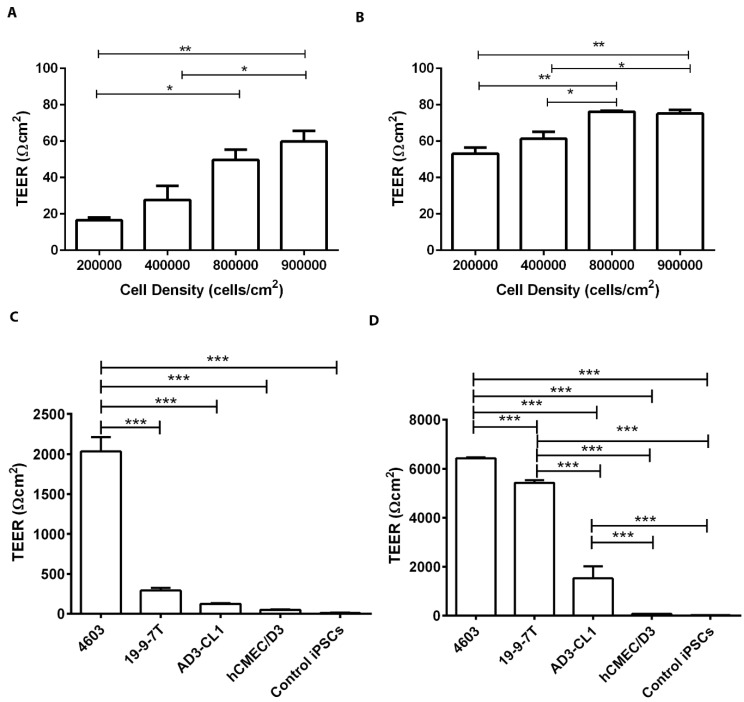
Optimisation of cell seeding density for TEER, and TEER measurement at 24 h and 48 h. hCMEC/D3 cells were seeded at the indicated densities on collagen-coated polyester Transwell^®^ inserts with 0.4 µm pore size. TEER measurements were performed at 24 h (**A**) and 48 h (**B**) post-seeding. TEER values of collagen I-coated inserts were subtracted from TEER measurements of hCMEC/D3. The maximum TEER value obtained was 80 Ωcm^2^, with 800,000 cells/cm^2^ at 48 h post-seeding. (**C**,**D**) TEER measurements in iPSC-derived BMECs and control cells. TEER of three different iPSC-derived BMECs, hCMEC/D3 cells and undifferentiated iPSCs (4603) was determined 24 h (**C**) and 48 h (**D**) post-seeding on Transwell^®^ inserts. TEER of coated inserts was subtracted from values obtained from all cell models. The 4603 iPSC-derived BMECs produced the tightest BBB model at both time-points. For all experiments, triplicate inserts were used to calculate mean ± SEM and statistical significance was calculated using one-way ANOVA, * *p* < 0.05, ** *p* < 0.01, *** *p* < 0.001.

TEER values of hCMEC/D3 have been reported to be between 30 and 50 Ωcm^2^ for monolayers under static conditions and around 1000 Ωcm^2^ under dynamic flow, but the TEER can rapidly decrease when flow is discontinued [6]. hCMEC/D3 cells were seeded at various densities on collagen I-coated cell culture inserts to investigate the optimum seeding density giving the highest TEER value at 24 h (Figure 2A) and 48 h (Figure 2B) post-seeding, after which TEER values decreased. TEER positively correlated with seeding density 24 h post-seeding, reaching 60 Ωcm^2^. At 48 h, TEER values positively correlated with cell density up to 800,000 cells/cm^2^, reaching a maximum of around 80 Ωcm^2^ (Figure 2B). For either time-point, there was no significant difference between TEER values obtained for densities of 800,000 and 900,000 cells/cm^2^; therefore, the latter density was used thereafter. The TEER of iPSC-derived BMECs and undifferentiated iPSCs was compared with that of hCMEC/D3 cells 24 h and 48 h post-seeding on Transwell^®^ inserts. The TEER of 4603-derived BMECs was significantly higher than those of all the other cell types at 24 h, reaching around 2000 Ωcm^2^ (Figure 2C). At 48 h, TEER values had increased considerably for all iPSC-derived BMECs. The TEER of 4603-derived BMECs was again highest at over 6000 Ωcm^2^, while that of 19-9-7T-derived BMECs was over 5000 Ωcm^2^ (Figure 2D). AD3-CL1-derived BMECs had lower TEER at around 1500 Ωcm^2^, but this was still about 20-fold higher than in hCMEC/D3. The TEER values of hCMEC/D3 cells were not significantly different to those of undifferentiated iPSCs. These results suggest that all three iPSC-derived BMECs form significantly tighter barriers than hCMEC/D3 and that the tightness of the barrier depends on the iPSC clone and time post-seeding.

### 3.3. Evaluation of Permeability of the BBB Models to Fluorescent Tracers

**Figure 3:**
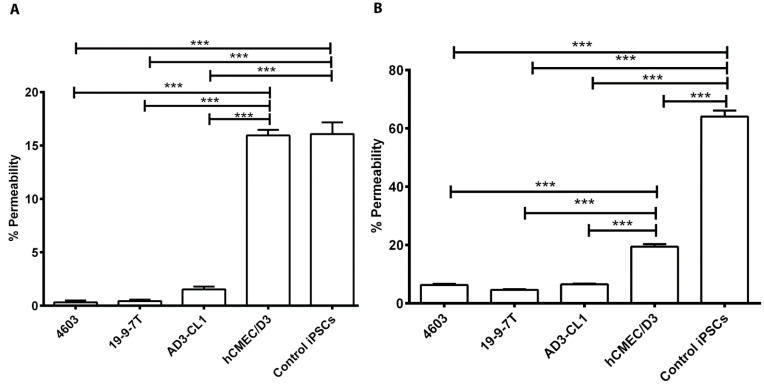
Permeability of iPSC-derived BBB models to para-cellular tracers. Three iPSC-derived BMECs, hCMEC/D3 and undifferentiated iPSCs (4603) were seeded on Transwell^®^ inserts, and 24 h later LY (**A**) or Na-F (**B**) was added to the apical chamber. Permeability is expressed as the percentage of tracer present in the basal chamber 1 h after administration. For all experiments, triplicate filters were used to calculate mean ± SEM. Statistical significance was calculated using one-way ANOVA. *** *p* < 0.001.

Permeability to para-cellular tracers is another way of assessing the tightness of the BMEC barrier, and lucifer yellow (LY) and sodium fluorescein (Na-F) are widely used fluorescent tracers with molecular weights of 550 Da and 376 Da, respectively. iPSC-derived BMECs and undifferentiated iPSCs were seeded on collagen IV and fibronectin-coated Transwell^®^ inserts at a density of 900,000 cells/cm^2^ and cultured for 48 h. hCMEC/D3 were seeded on collagen I-coated Transwell^®^ inserts at the same density and maintained for 48 h. LY or Na-F was then added to the apical chamber and buffer was added to the basal chamber. Cells were incubated at 37 °C for 1 h and samples from the basal chamber were withdrawn and analysed on a fluorescent plate reader. Less than 2% of LY passed through iPSC-derived BMECs, whereas permeability through hCMEC/D3 and undifferentiated iPSCs was similar at around 16% (Figure 3A). A similar pattern was seen for the permeability to Na-F through iPSC-derived BMECs, but at higher levels of about 5% (Figure 3B). Permeability to Na-F through hCMEC/D3 was significantly higher than in iPSC-derived BMECs but significantly lower than that of control iPSCs. As expected, permeability to Na-F was higher than to LY because the latter has higher molecular weight. These results, taken together, again suggest that the BBB models formed by iPSC-derived BMECs are significantly tighter than the hCMEC/D3 system.

### 3.4. Analysis of BBB Model Crossing by AAV8 and AAV9 Vectors and Cell-Penetrating Peptide-Conjugated PMO

**Figure 4:**
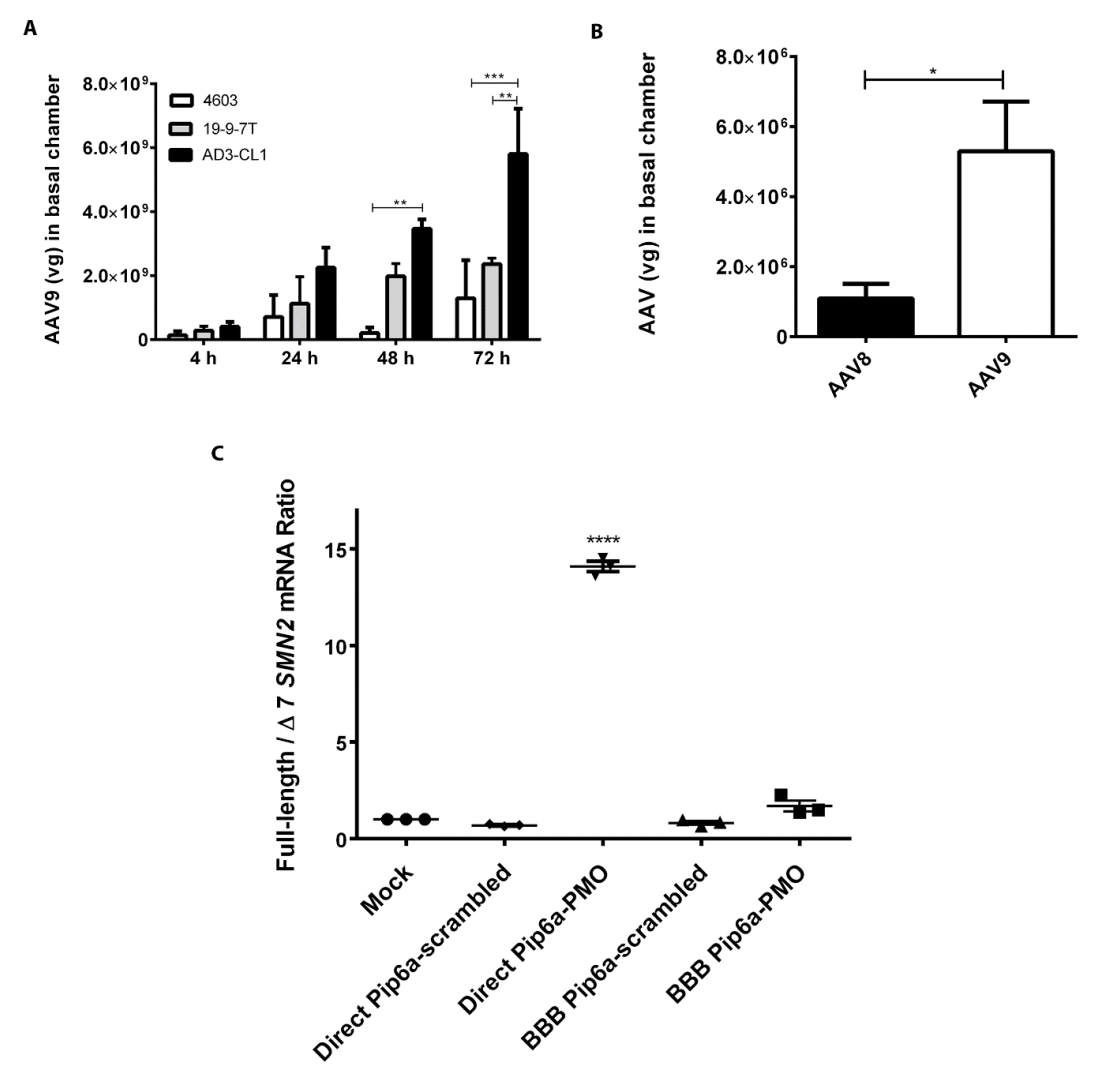
Crossing of AAV vectors and cell-penetrating PMO through iPSC-derived BMEC BBB models. (**A**) Three iPSC-derived BMEC models were seeded on Transwell^®^ inserts and 7.7 × 10^10^ vg units of ssAAV9-CBA-eGFP were added to the apical chamber. Sampling was performed in the basal chamber at the indicated time-points. Triplicate experiments were used to calculate mean ± SEM, and statistical significance was calculated using two-way ANOVA, ** *p* < 0.01, *** *p* < 0.001. (**B**) A comparison of AAV8 and AAV9 vector crossing of BBB formed by 4603-derived BMECs. 7.7 × 10^10^ vg units of ssAAV8-Spc512-eGFP or scAAV9-CMV-eGFP were added to the apical chamber. Sampling was performed in the basal chamber at 72 h. All experiments were carried out at least in triplicate to calculate mean ± SEM, and statistical significance was calculated using t test, * *p* < 0.05. (**C**) Crossing of Pip6a-PMO across BBB formed by 4603-derived BMECs. In this system, effective treatment with Pip6a-PMO would alter the full length to ∆7 *SMN2* mRNA ratio in the target type I SMA fibroblasts in the basal chamber. As a positive control (labelled “Direct”), PMO was added directly to the SMA type I fibroblast cells in the basal chamber at 4-fold lower concentration. To test the PMO penetration through the BBB, PMO was added to the apical chamber of the Transwell^®^ inserts seeded with BMECs (labelled “BBB”). Pip6a-scrambled PMO was used as a negative control. qRT-PCR quantification of *SMN2* mRNA transcripts in SMA type I fibroblasts was performed. Data were normalised to the housekeeping gene *GAPDH*, standardised to levels in mock fibroblasts and expressed as full-length to ∆7 *SMN2* mRNA ratios. For all experiments, triplicate samples were used to calculate mean ± SEM, and statistical significance was calculated using one-way ANOVA, **** *p* < 0.0001.

As AAV9 vectors have been shown to cross the BBB in several in vivo studies involving different animal models and humans following intravascular delivery [20,21,26,27], we investigated AAV9 vector crossing through our three iPSC-derived BMEC models of the human BBB (Figure 4A). To test the system, BMECs differentiated from iPSCs were seeded as stated and 7.7 × 10^10^ vg particles of ssAAV9-CBA-eGFP were added to the apical chamber of the Transwell^®^. Medium samples were harvested from the basal chamber at 4, 24, 48 and 72 h. Vector genomes were quantitated using qPCR in the basal chamber samples. We observed crossing of the AAV9 vector in a BMEC clone- and time-dependent manner. AAV9 showed crossing capability in all three models, with a maximum of around 5.8 × 10^9^ vg at 72 h in AD3-CL1-derived BMECs (Figure 4A). The lowest level of AAV9 crossing at 72 h was around 1.3 × 10^9^ vg, observed in the tightest model, 4603-derived BMECs.

As 4603-derived BMECs showed to be the most challenging model for AAV9, we further compared AAV8 and AAV9 vector crossing in this model (Figure 4B). These vectors were produced and purified in house by the same method for this study. 7.7 × 10^10^ vg units of ssAAV8-Spc512-eGFP or scAAV9-CMV-eGFP vectors were added to the apical chamber of the Transwell^®^. Comparison of AAV8 and AAV9 crossing at 72 h revealed that 5.3 × 10^6^ vg AAV9 crossed the 4603-derived BBB while only 1.1 × 10^6^ vg of AAV8 crossed. AAV9 showed higher crossing capability in the first experiment (Figure 4A) compared to the second (Figure 4B), possibly reflecting differences between commercial and in-house produced vector, and the different qPCR detection methods used in the two experiments.

We further investigated BBB crossing by another type of genetic treatment for SMA, a cell-penetrating peptide-conjugated morpholino. A high concentration of systemically administered Pip6a-PMO able to promote the inclusion of *SMN2* exon 7 (hereby named Pip6a-PMO) has shown positive effects in a severe SMA mouse model [19]. Therefore, we investigated the BBB-crossing ability of Pip6a-PMO in the tightest iPSC-derived BBB model (4603). The model was set up as for the previous experiments with AAV, but indicator SMA type I fibroblasts were seeded on the basal chamber. PMOs (either Pip6a-PMO or a control Pip6a-scrambled PMO) were added to the apical side or directly to the target type I SMA fibroblasts. When PMO was added directly to type I SMA fibroblasts, the concentration was 4-fold lower to compensate for the dilution that PMOs crossing the BBB would undergo when added to the apical chamber. The SMA type I fibroblasts from the basal chamber were harvested 24 h after PMO treatment, RNA was extracted, cDNA was synthesised and used in qPCR to analyse the ratio of full-length to ∆7 *SMN2* mRNA (Figure 4C). The addition of the Pip6a-scrambled PMO had no effect on the mRNA ratio. Direct treatment of fibroblasts with Pip6a-PMO led to a 14-fold increase in the ratio, as expected from this positive control. However, although the addition of the Pip6a-PMO to the apical chamber of the BBB model led to a slight increase in the full-length to ∆7 mRNA ratio when compared to mock or Pip6a-scrambled PMO-treated fibroblasts, the increase was not statistically significant. These results suggest that AAV9 vector can efficiently cross our tightest model of the human BBB, while AAV8 and Pip6a-PMOs cannot.

## 4. Discussion

Efforts to model the BBB in vitro have included the use of primary animal BMECs, primary human BMECs from biopsies, co-cultures of BMECs with different combinations of other BBB cells, and commonly, the immortalised human cerebral microvascular endothelial cell line hCMEC/D3. Animal cell-derived systems are not ideal to model the human BBB and the use of primary human cells is not sustainable. The use of hCMEC/D3 cells was a significant advance, but this cell line has several limitations, such as low TEER and reduced expression of tight junction proteins [7]. The development of iPSC technology has allowed the generation of numerous human cell lineages in culture, both wild-type and disease-specific. This is of particular importance for cell types that cannot be easily sourced, like BMECs and motor neurons. The availability of a reliable protocol for differentiation of iPSCs into BMECs has enabled the successful production of human models of the BBB. We have used three different wild-type iPSC clones (4603, 19-9-7T and AD3-CL1) and included hCMEC/D3 as a benchmark control. The iPSC-derived BMEC lines were characterised using immunocytochemistry for expression of tight junction proteins occludin and claudin–5, flow cytometry for GLUT–1 and PECAM–1, TEER, and permeability to para-cellular tracers LY and Na-F. Then, these BBB models were used to test BBB crossing by AAV8 and AAV9 vectors, and peptide-conjugated PMOs.

The maximum in vitro TEER value reported to date is around 8000 Ωcm^2^, using iPSC-derived BMECs in serum-free medium including B27 supplement [28]. The average TEER of microvascular vessels in the frog brain was calculated to be around 2000 Ωcm^2^ [29] whereas the TEER of arterial vessels of young rats was reported to be around 1500 Ωcm^2^ and venous vessels around 900 Ωcm^2^ [30]. The highest in vivo TEER was calculated to be around 8000 Ωcm^2^ for rats [31]. A TEER of 6600 Ωcm^2^ has previously been reported for a co-culture system involving iPSC-derived BMECs, pericytes and astrocytes [32]. The maximum TEER reported to date for an in vitro microfluidic co-culture system is around 4500 Ωcm^2^ [10]. A recent 4-cell in-vitro blood–brain barrier model was reported to have a maximum TEER of 230 Ωcm^2^ [33]. In our experiments, TEER of 4603-derived BMECs was above 6000 Ωcm^2^, over 70-fold higher than in hCMEC/D3 cells, while for 19-9-7T-derived BMECs it was over 5000 Ωcm^2^. TEER values of AD3-CL1 BMECs were lower, at around 1500 Ωcm^2^.

Furthermore, 4603 and 19-9-7T BMECs were also consistently more proficient than AD3-CL1 BMECs in relation to other features of the BBB, including expression of occludin and claudin–5, and low permeability to para-cellular markers. hCMEC/D3 was less efficient in all these tests, suggesting that the TEER of all these iPSC-derived BBB models correlates well with other BBB features and depends on the iPSC clone they were differentiated from. Inter-individual variation in cell maturation and functionality of iPSC-derived BMECs has been previously reported [34]. In our experience, further differences between iPSC-derived BMECs and hCMEC/D3 include the very low percentage of cells expressing GLUT–1 in the latter, while about 20% of iPSC-derived BMECs were positive for it. The opposite is true for the production of PECAM–1, positive in around 66% of hCMEC/D3 cells, whereas only 0.3–3% of iPSC-derived BMECs display it. The available literature is inconsistent regarding GLUT–1 levels in hCMEC/D3, and both presence [35] and absence [8] have been reported. It has also been reported that the expression of PECAM–1 is low in human brain tissue [36]. We postulate that the shear stress caused by the flow and exposure to platelets and other cells in blood is important for the expression of PECAM–1. The hCMEC/D3 is a primary human cell line that has been exposed to blood flow and that could be the reason for the relatively high expression of PECAM–1 in hCMEC/D3 compared to iPSC-derived BMECs that have never been exposed to fluid flow.

Many serotypes of AAVs have been tested for proficiency to cross the BBB [37]. Facial vein injection of AAV9-GFP into neonatal mice has resulted in extensive transduction of spinal cord and brain neurons [20]. It has also been reported that AAV9 penetrates a primary human BMEC BBB model more effectively than AAV2 [38]. We investigated BBB crossing by AAV8, and also by two different AAV9 stocks produced in different settings, over a time period between 4 and 72 h. The crossing of commercially produced AAV9 increased in a time-dependent manner to a maximum of around 5.8 × 10^9^ vg at 72 h. Crossing was greatest through AD3-CL1-derived BMECs and lowest through 4603-derived BMECs. This suggests an inverse correlation between the tightness of the barrier and AAV9 crossing, indicating the involvement of para-cellular transport of AAV as well as other mechanisms such as transcytosis [39,40]. Direct comparison of AAV8 and AAV9 produced in house revealed 5-fold more AAV9 crossing after 72 h compared to AAV8, but less crossing compared to commercially produced AAV9.

Cell-internalising peptide-conjugated PMOs have been designed to improve cell uptake of PMOs in several diseases, including spinal muscular atrophy [17,18]. Systemically administered Pip6a-PMO has been shown to have positive effects in severe SMA mice [19], but the BBB is reported to be much tighter in humans compared to rodents [30,31]. We investigated the crossing of Pip6a-PMO through 4603 BMECs, our tightest BBB model. Although direct treatment of SMA type I fibroblasts with Pip6a-conjugated PMO elevated their full-length/∆7 *SMN2* mRNA ratio, Pip6a-PMO added to the BBB model did not have any significant effect on full-length/∆7 *SMN2* mRNA ratio in SMA type I fibroblasts in the basal chamber, indicating no BBB crossing. It should be noted that when assessing BBB crossing of gene therapies such as AAV vectors and antisense oligonucleotides, the potential effect of these therapeutics on the BMECs should be considered. These drugs could affect the phenotype of BMECs, for example, by altering the expression of BBB junction and transporter proteins, thus possibly modifying TEER values and permeability of the BBB.

The purpose of this study was to assess several iPSC-derived BMEC lines for the generation of simple and reproducible models of the BBB, and to compare them to the most widely used and characterised human in vitro model, hCMEC/D3, with the ultimate goal of testing therapeutics. All three iPSC-derived BBB models were superior to hCMEC/D3, and the quality of the barrier was dependent on the iPSC clone used. Our study demonstrates that hCMEC/D3 has reduced expression of tight junction proteins and GLUT–1, which is in line with the findings reported after transcriptional profiling [8]. hCMEC/D3 retains some properties of endothelial cells, including the expression of PECAM–1, but displays significant reduction in some properties of brain endothelial cells, such as the ability to form tight junctions, as detected using TEER and immunocytochemistry. We have demonstrated that BBB models using iPSC-derived BMECs alone can exhibit very tight junctions, comparable to BBB in vivo, without the need for complicated co-culture systems. In particular, 4603-derived BMECs displayed TEER values comparable to those obtained in co-culture systems [12], making this a simple, tight, scalable and reproducible model of the human BBB, with many potential uses, including high-throughput testing of potential therapeutic agents.

## Data Availability

The datasets that were generated and/or analysed during the presented study are available from the corresponding author upon reasonable request.
